# Determinants and Outcomes of Decision-Making, Group Coordination and Social Interactions during a Foraging Experiment in a Wild Primate

**DOI:** 10.1371/journal.pone.0053144

**Published:** 2013-01-10

**Authors:** Lennart W. Pyritz, Claudia Fichtel, Elise Huchard, Peter M. Kappeler

**Affiliations:** 1 Behavioral Ecology and Sociobiology Unit, German Primate Center, Göttingen, Germany; 2 Courant Research Centre “Evolution of Social Behaviour”, University of Göttingen, Germany; Institut Pluridisciplinaire Hubert Curien, France

## Abstract

Social animals have to coordinate joint movements to maintain group cohesion, but the latter is often compromised by diverging individual interests. A widespread behavioral mechanism to achieve coordination relies on shared or unshared consensus decision-making. If consensus costs are high, group fission represents an alternative tactic. Exploring determinants and outcomes of spontaneous group decisions and coordination of free-ranging animals is methodologically challenging. We therefore conducted a foraging experiment with a group of wild redfronted lemurs (*Eulemur rufifrons*) to study decision outcomes, coordination of movements, individual foraging benefits and social interactions in response to the presentation of drinking platforms with varying baiting patterns. Behavioral observations were complemented with data from recordings of motion detector cameras installed at the platforms. The animal's behavior in the experimental conditions was compared to natural group movements. We could not determine the type of consensus decision-making because the group visited platforms randomly. The group fissioned during 23.3% of platform visits, and fissioning resulted in more individuals drinking simultaneously. As under natural conditions, adult females initiated most group movements, but overtaking by individuals of different age and sex classes occurred in 67% of movements to platforms, compared to only 18% during other movements. As a result, individual resource intake at the platforms did not depend on departure position, age or sex, but on arrival order. Aggression at the platforms did not affect resource intake, presumably due to low supplanting rates. Our findings highlight the diversity of coordination processes and related consequences for individual foraging benefits in a primate group living under natural conditions.

## Introduction

Group-living holds a number of benefits, but also costs for individual group members. Many of the benefits, such as shared vigilance or predator confusion, are related to reduced per capita predation risk, whereas increased intra-group feeding competition represents one of the unavoidable main costs of group-living [1]–[3]. Cohesion is a prerequisite for reaping the major benefits of group-living, and consensus decisions enable group members to achieve cohesion by coordinating their activities and travel schedules [4]–[6]. However, joint decision-making can be hampered by diverging individual needs due to differences in sex, age, motivation as well as reproductive and physiological state among group members [7]. Group-living animals can nevertheless reach a consensus via a continuum of decision-making processes, depending on the proportion of group members involved in the decision. In equally shared consensus decisions, each individual has the opportunity to influence the outcome of a decision in a voting process. In unshared consensus decisions, only one individual decides, e.g. when to change place and activity, irrespective of other individuals' interests, and all group members abide by this decision. Between these two extremes, consensus decisions can be more or less partially shared [4], [8].

Group fission, i.e. temporary splitting of a group into two or more subgroups, has often been interpreted as the outcome of incompatible individual interests [4], [9]. Thus, fissioning may allow group members to avoid costly consensus decisions under certain conditions without foregoing the benefits of group-living for a long time [10]. For instance, fissioning has been observed in homing groups of domestic pigeons (*Columba domestica*: [11]) and in Bechstein's bats (*Myotis bechsteinii*) switching communal roosts [12]. In a recent field experiment, temporary splitting into subgroups has been observed in a troop of chacma baboons (*Papio ursinus*) visiting small artificial food patches when followers had weak social ties with the dominant male leading the group [13]. Short-term group fission also occurs in Tonkean macaques (*Macaca tonkeana*) and rhesus macaques (*Macaca mulatta*) [14].

Most empirical and theoretical studies explore decision-making and group cohesion in the context of group movements because they provide a biologically meaningful model and uniform context to study determinants and consequences of relevant coordination processes (e.g., modelling: [7]; rhesus and Tonkean macaques: [15], [16]; domestic geese, *Anser domesticus* and heifers, *Bos taurus*: [17], [18]; meerkats, *Suricata suricatta*: [19]). In order to operationalize and quantify the coordination of group movements, it has proven useful to divide them into successive phases that are defined taxon-specifically to determine which individuals initiate, whether the leader is overtaken, who terminates the movement, how many group members follow in which time frame, and how far the group travels [20], [21].

In the context of studying the behavioral mechanisms structuring group movements, a leader can be defined as an individual that exerts social influence on fellow group members and elicits follower behavior [22]–[25]. Leadership can be distributed over (a subset of) all group members, or one individual can lead the group consistently [4], [13], [26]. The definition of a leader should not focus on its spatial position during a group movement because individuals may also lead from behind, i.e. initiate and terminate a movement without being at the forefront of the group [24]. However, in this study we use the term leader for the individual moving at the head of the group or arriving first at a given destination (in this case: foraging platforms) because we focus on consequences of the spatial position during movements on foraging benefits. Leadership during group movements in this sense is considered to be stable if overtaking occurs rarely during travelling, and unstable if the leading individual often changes within one single travel event [24].

While it is already challenging to observe and analyse coordination processes of animal group movements in their natural habitat, it is even more difficult to determine proxies of fitness consequences of the underlying decisions for single group members, such as individual foraging benefits [21]. Experimental approaches have therefore been implemented in studies of coordination and decision-making regarding collective movements in various taxa (house-hunting rock ants, *Temnothorax albipennis*: [9]; honey bees, *Apis mellifera*: [27]; sticklebacks, *Gasterosteus aculeatus*: [28]; domestic pigeons: [11]; Bechstein's bats: [12]; white-faced capuchin monkeys, *Cebus capucinus*: [29]; meerkats: [30], chacma baboons: [13]). In some of these experiments, group decisions regarding movements to experimental feeding patches were studied. For example, field experiments with wild capuchin monkeys (*Cebus apella nigritus*) focused on food detection abilities depending on distance, travel speed and resource size [31], spatial memory and strategic route planning depending on varying baiting patterns of the platforms [32], [33], or individual foraging benefits through deceptive vocalisation (predator alarm calls) at feeding platforms [34]. Experiments in captivity revealed that the spatial decisions of capuchin monkeys to move towards two mangers installed in different areas of their enclosure were mainly driven by anonymous mimetism, i.e. individuals tended to follow the travel routes previously taken by their group mates [29]. A similar experiment with free-ranging chacma baboons revealed that the dominant males initiated and led all group movements to artificial feeding patches and also acquired the greatest foraging benefits. Subordinates followed the leading male in most cases despite considerable consensus costs [13]. Little is still known about behavioral tactics that could decrease consensus costs, e.g. fissioning or overtaking, and how these tactics affect individual foraging success, however.

In the present study, we experimentally investigated decision outcomes, coordination processes, individual foraging benefits and the proximate mediation of conflict in a group of wild redfronted lemurs (*Eulemur rufifrons*). Redfronted lemurs provide an interesting model in this context for a number of reasons. First, they live in small egalitarian groups with rather equally distributed resource-holding potential [35], [36], providing an interesting contrast in traits influencing leadership and decision-making to taxa with larger groups or with pronounced dominance hierarchies (e.g., dwarf mongooses, *Helogale parvula*: [37]; plains zebras, *Equus burchellii*: [38]; chacma baboons: [13]). Second, our study group is free-ranging and co-resides with a number of different predator species [39], [40], which should favor group cohesion and consensus decision-making [41]. Finally, group-living evolved at least twice independently among the primates of Madagascar, compared to only once among the ancestral anthropoids [42], on which most current primate coordination studies have been conducted (summarized in [43]). Thus, lemurs can provide important comparative information on the convergence of group coordination in primates from an evolutionary perspective.

We pursued two main goals with this study: (1) to measure the extent to which decisions are shared, and (2) to explore the determinants and efficiency of three potential tactics to decrease the consensus costs of unshared or partially shared decisions, including (i) group fission during group movement, (ii) overtaking during group movement, and (iii) aggressive supplants once the final destination is reached.

We first explored the extent to which decisions were shared. To do so, we set up drinking platforms and created conflicts of interest of varying degrees by modifying baiting patterns of the platforms in two different designs, i.e. providing either several group members or only a single individual with drinking opportunities (details below). Our predictions regarding the type of consensus decision-making at departure referred to the decision outcomes, i.e. the observed distribution of the group between the experimental platforms (Table 1): We hypothesized that unshared consensus decision-making would predominantly result in one individual leading the group to a platform that guarantees maximum resource intake for itself, irrespective of foraging opportunities for followers. In contrast, shared decision-making was expected to result in the group preferably visiting platforms offering rewards for several individuals.

**Table 1:** Experimental designs and respective predictions for different types of consensus decision-making.

Condition	Platform	No. of bottles	Vol. Per bottle (ml)	Expected decision types for different decision outcomes
**Start of data collection (Design 0)**				Habituation (Data included in tests on resource intake, group fissioning and aggressiveness.)
Condition 1: Aug 4–7	1	1	75	
	2	1	75	
	3	5	75	
	4	1	75	
**Design 1**				*Shared consensus decision-making:* Group visits platforms baited with 5 bottles more often than expected randomly. *Unshared consensus decision-making:* Group visits platforms baited with 1 bottle more often than expected randomly.
Condition 2: Aug 11–14	1	1	75	
	2	1	75	
	3	5	30	
	4	1	75	
Condition 3: Aug 18–21	1	1	75	
	2	5	30	
	3	1	75	
	4	1	75	
**Design 2**				*Shared consensus decision-making:* Group visits platform baited with 5 bottles more often than expected randomly. *Unshared consensus decision-making:* Group visits platform baited with 1 bottle more often than expected randomly. Test effects of the number of baited platforms on group fissioning and aggressiveness
Condition 4: Aug 25–28	1	-	-	
	2	5	10	
	3	1	30	
	4	-	-	
Condition 5: Sep 1–4	1	-	-	
	2	1	30	
	3	5	10	
	4	-	-	

We also explored the circumstances favoring group fissioning, predicting group fission rates – as a means to avoid costly consensus decisions in the first place by foraging in independent subgroups – (i) to decrease when the number of baited platforms was reduced, and (ii) to increase after the group visited a poorly baited platform where only one group member has access to a valuable resource instead of five (details on baiting patterns below). The efficiency of group fission in decreasing consensus costs was measured by comparing the number of individuals accessing foraging rewards simultaneously in the presence versus in the absence of group fission. We expected group fission (iii) to result in more individuals drinking at the same time.

Third, we explored individual and ecological factors favoring overtaking during group movements. Redfronted lemurs are characterized by stable leadership of several adult females per group under natural conditions [44], [45]. We therefore expected adult females to predominantly initiate and lead group movements and to have the highest resource intake at the drinking platforms. In addition, we expected overtaking ( = change of leadership) rate to be higher during movements towards one-bottle-platforms, because they only reward the first individual arriving. The efficiency of overtaking in compensating consensus costs was investigated by measuring the impact of departure and arrival order on individual resource intake at the platforms.

Finally, we explored the circumstances favoring aggressiveness at the platforms – as a way to increase foraging benefits irrespective of coordination during group movements –, expecting its frequency to be negatively correlated with the number of baited platforms, which is here supposed to reflect overall resource abundance and thus the intensity of within-group feeding competition [46]. The efficiency of aggression in compensating consensus costs was measured by comparing individual resource intake after emission of aggression versus in the absence of aggression.

## Methods

### Study site and subjects

Data were collected at the field station of the German Primate Center (DPZ) at Kirindy Forest, a dry deciduous forest located about 60 km north of Morondava, western Madagascar [47]. The site is managed within a forestry concession operated by the Centre National de Formation, d'Études et de Recherche en Environnement et Foresterie (CNFEREF), Morondava. The forest is characterized by a pronounced seasonality with a hot wet season between December and March and a cooler dry season between April and November [48]. Redfronted lemurs at Kirindy face a number of predators, including fossas (*Cryptoprocta ferox*), Harrier hawks (*Polyboroides radiatus*), stray dogs (*Canis familiaris*) and Malagasy boas (*Acranthrophis ssp.*) [39], [40].

**Figure 1:**
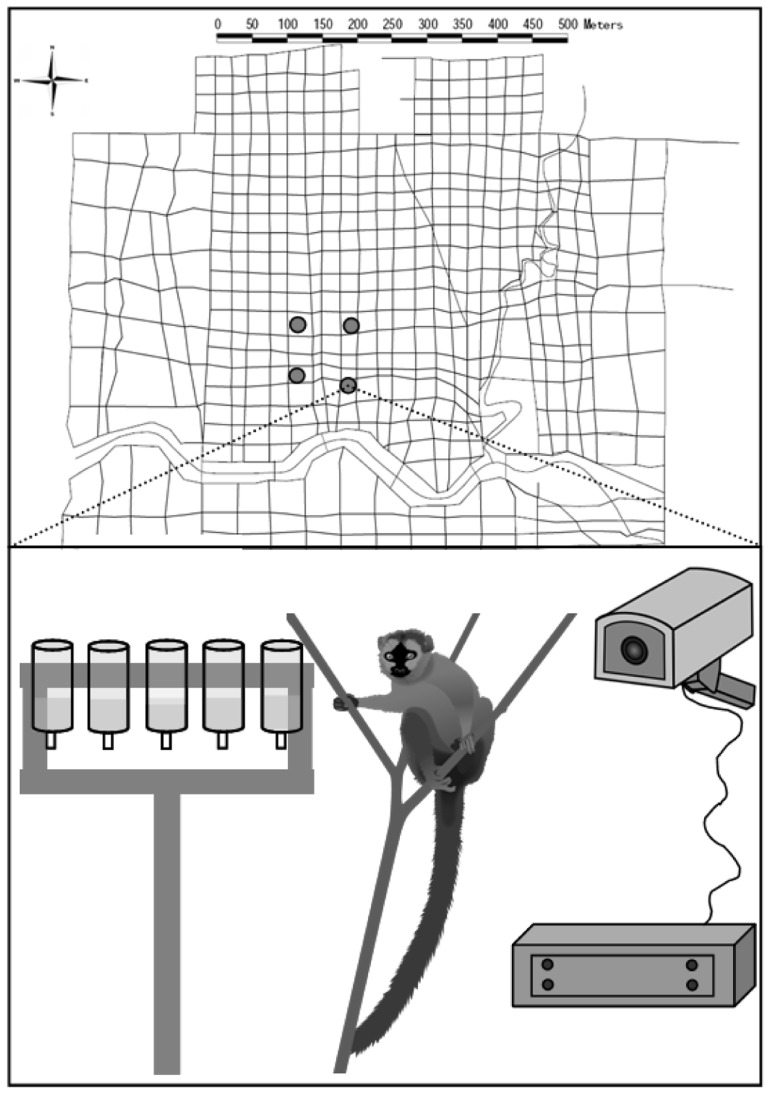
Trail system of the study area at Kirindy Forest with locations of the 4 drinking platforms (above). Experimental setup: Platform with (a maximum of) 5 drinking bottles, monitored by a motion detector camera connected to a digital recorder (below).

One group of redfronted lemurs (“group B”) regularly visited the drinking platforms (see below). It consisted of 12 individuals (3 adult females, 5 adult males, 1 subadult female, 1 subadult male, 2 juvenile males; adults >2.5 years, subadults 1–2.5 years, juveniles <1 year). The study group is part of a habituated population inhabiting a 60-ha study area. The study area features a grid system of foot trails with intersections every 25 m (Figure 1). The members of the study group have been regularly captured, individually marked and observed since 1996 (e.g., [49]). Kin relations were known for all dyads ([50], [51], Kappeler et al. unpub. data), except those involving 3 adult male immigrants from 2008 (BMNeg, BMPan, BMRot). Research presented in this manuscript was authorized by the Département de Biologie Animale, Université d'Antananarivo, the CAFF of the Direction des Eaux et Forêts and the CNFEREF Morondava. The official permit number from the Malagasy Ministère de l'Environnement, des Forêts et du Tourisme is 51/09/MEFT/SG/DGEF/DSAP/SLRSE (Renouvellement de l'Aut No 213/08 du 28/08/2008). In contrast to countries like the USA, neither Germany nor Madagascar do have or require a statement from an ethical or animal welfare committee. Implicit assessment of this aspect of research projects is included in the decision of legal authorities that authorize research in the respective country.

Data were collected during the peak dry season (August-September) in 2009, when most trees had shed their leaves and only a few small water holes remained in the nearby Kirindy river bed (curved line in Figure 1). During this time of year, several groups of redfronted lemurs make daily forays of up to 2 km to these water holes [52], demonstrating that water represents a high-value resource for them. Redfronted lemur reproduction is highly seasonal, with mating taking place in May/June and births in September/October [53]. The adult females were therefore pregnant during this experiment.

### Experimental setup and designs

We tested the experimental setup in a pilot study on 6 days between June-August 2009 with a group of redfronted lemurs that regularly visited the research camp. We attached 1 to 5 small drinking bottles (maximum volume per bottle = 250 ml) in bushes in the camp area in close proximity to each other (approx. 15 cm). The bottles contained different volumes (10–100 ml) of sweet grenadine syrup and water in a 1∶10 ratio and had a light red colour. As soon as the lemurs had realized that the bottles provided drinking opportunities, they usually moved towards them quickly and started drinking as soon as they were attached. Due to this behavior, we considered the value of flavored water as an experimental resource as high, especially because there were other water sources available. During the pilot study we observed in several cases that (i) redfronted lemurs were obviously able to distinguish between different syrup volumes and that they preferably approached bottles containing larger volumes. We never observed (ii) single individuals monopolizing several bottles that were positioned in close proximity, or (iii) individuals displacing conspecifics from other bottles after depleting their own one. We found that single lemurs always emptied volumes of 10 ml. Bottles containing 30 ml were depleted in approx. 70% of cases. In contrast, bottles containing ≥75 ml were only emptied in approx. 50% of cases. Thus, we considered 10 ml as small, 30 ml as medium and 75 ml as large volumes in the experiment (Table 1).

For the actual data collection, we set up 4 drinking platforms placed 75 m apart from each other in the core area of group B's home range (Figure 1). We carefully chose the positions of the platforms to be as similar as possible, i.e. similar position between larger trees, no feeding trees in direct vicinity, similar shrub density etc. In non-experimental observations of movements in a total of 4 groups of redfronted lemurs between 2007 and 2010, all members of a group were usually detected within a 20 m radius (88.8% of approx. 7600 scans of spatial group distribution). Usually, 57.6% of group members even gathered within a 10 m radius [45]. There are no data on group spread >20 m in this species. However, based on the distance scans in [45] and personal observations by PMK, CF, LWP as well as local field assistants we consider the distance of 75 m between the platforms as clearly more than the usual group spread and we define a fission event as individuals from the same group being ≥ approx. 75 m away from each other. Thus, the group had to fission in order to exploit two platforms simultaneously. Each foraging station consisted of a wooden platform with a maximum of 5 drinking bottles attached to it. Each drinking platform (including the surrounding area with a radius of approx. 3 m) was monitored constantly by a custom-made surveillance system including a motion detector camera connected to a digital recorder in a waterproof box (Neumann, Ettlingen, Germany; Figure 1). Footage of lemur visits at the platforms could be downloaded in the forest with a portable monitor and remote control. Note that, with this method of data collection, not all individuals that were present close to a platform were necessarily visible on the recordings.

After a 2-week habituation phase in which all platforms were provided with 5 bottles (each containing 75 ml) every day, the study group visited all 4 platforms regularly, and we started data collection using the camera systems (Table 1). In the first condition of the experiment (Design 0), we did not change the amount of water per bottle but provided only one bottle on platforms 1, 2 and 4 in order to habituate the group to varying numbers of bottles. Data collected during this condition were not used to explore the decision type because unshared decision-making was not expected to lead to particular platforms due to an equal amount of resource in all bottles. However, data collected in condition 1 were included in tests of resource intake, group fission and aggressiveness.

Design 1 started with condition 2 (Table 1). Here, we additionally reduced the amount of water in the 5 bottles on platform 3 to 30 ml. Under these conditions, we predicted shared decision-making to result in more frequent visits to platform 3 than expected randomly because it offered drinking opportunities for 5 group members. Conversely, unshared decision-making should bias visit rates towards the other platforms, baited each with one bottle containing 75 ml. Condition 3 equalled condition 2, but baiting patterns of platforms 2 and 3 were reversed to exclude habituation effects to platform 3.

Condition 4 marked the beginning of design 2. On the one hand, we reduced the number of baited platforms from 4 to 2 in order to explore the consequences for group fissioning and aggressiveness at the platforms. Additionally, we reduced the resource amount at the 5-bottle-platform from medium (30 ml) to small (10 ml) and at the remaining 1-bottle-platform from large (75 ml) to medium (30 ml). We conducted these changes because we observed in the pilot study that only half of the individuals depleted 75 ml at once, i.e. a platform baited with only 1 bottle containing 75 ml could possibly offer drinking opportunities for at least 2 individuals, whereas bottles containing 30 ml were depleted by 1 individual in 70% of cases. Thus, we aimed at sharpening the conflict among group members when making consensus decisions. As in design 1, we expected shared decision-making to result in preferred visits to the 5-bottle-platform and unshared decision-making to result in visits to the 1-bottle-platform. Condition 5 equaled condition 4 but the baiting patterns of platforms 2 and 3 were shifted to control for habituation effects.

Each condition lasted for 1 week. However, data were recorded only during the last 4 days of each condition to give animals enough time to habituate to the new baiting pattern. During the experiment, all platforms were controlled every 30 min. If the focal group depleted the platforms in the morning, bottles were refilled once around 2 pm. If a group other than the focal group visited the platform and drank water, bottles were refilled according to the respective experimental condition. We also refilled bottles when the identity of the group that had depleted them before was unknown. Since this unknown group later turned out to be group B quite often, two or more visits of the group B at the same baited platform during one day occurred regularly (Table S1).

### Data collection

Data were collected between August 4 and September 4 (Table 1), with the camera system operating from 6:15–17:00 h, on each day of the experiment. All visits of the study group recorded during this time (n = 110; approximate total duration: 10 h) were analysed using EverFocus Player MFC Application 2008 (EverFocus Electronics Corporation, Emmerich, Germany). Data extracted from the videos included number of daily visits per platform, total number of individuals visiting the platform and individual length of stay, arrival order, individual resource intake and frequency of agonistic interactions at the platforms. Individual resource intake could be assessed accurately from the videos due to the light red colour of the flavoured water and highlighted measuring lines on the drinking bottles. For agonistic interactions (bite, chase >2 m, cuff, grab), each event was counted separately because one bite or cuff could possibly supplant an opponent from a bottle and offer a drinking opportunity. Based on the definition given above, a fission event was only counted if 1 or more individuals of the study group were recorded at different platforms at the same time. We also included visits of group members at different platforms that occurred <1 min after one another as a fission event (n = 4) because intervals between subsequent visits (on the same day: mean = 4.5±2.2) were usually much longer (max = 571 min, min = 0.38 min [23 sec], median = 14.9 min [894 sec]) and the shortest interval of the same individual being recorded at two different platforms was 64 s (BMSaw, August 14^th^, 2009, 15∶45∶43 at platform 2 and 15∶46∶47 at platform 1). Therefore, we consider 1 min as a suitable threshold to define a fission event at the platforms. Accordingly, subgroup size was calculated as the number of group members that were recorded at a platform while there were group members at a different platform at the same time or <1 min before or after.

Behavioral observations were also conducted during 17 days (85%) of the experiment (total of 81 h), usually by two observers (for details of the observation protocol see [45]). When we observed the group, we stayed with the animals between approximately 7:00 to 10:00 h and 14:00 to 17:00 h. During these observations LWP recorded every group movement according to an operational definition developed during a pilot study, and recorded details of initiation, followership and overtaking in a total of 25 movements that terminated at a drinking platform [21], [43], [45]. However, these movements often proceeded fast and with multiple change of leadership, making it difficult to protocol each detail. Therefore, we assessed overtaking events during most movements retrospectively by checking if the initiator of a movement also arrived first at the drinking platform on the corresponding video. If the initiator arrived ≥5 sec (usually corresponding to several body lengths) after the first individual at the platform, this was counted as an overtaking event that was comparable to the definition of overtaking used in movements that did not terminate at a drinking platform ([45]: “Overtaking occurred if a follower outdistanced the leader, i.e. the individual at the forefront of the group, by more than three body lengths without diverging more than approximately 45 degrees of the initial trajectory of travel.”). We did not record fission events during movements that did not terminate at a foraging platform. During these movements we could not determine inter-individual distances of group members that were greater than 20 m accurately. Thus, we could not use the same definition as for movements that terminated at platforms and observations would not have been comparable. However, as mentioned above, all members of a group were usually detected within a 20 m radius during observations not involving the foraging platforms.

### Data analyses

We used a Mann-Whitney U*-*test (MWU) to explore whether the number of bottles per platform (1 or 5) affected the number of individuals per visit, i.e. whether more bottles indeed provided more individuals with a foraging opportunity. Whether group fissioning was beneficial in terms of more individuals drinking simultaneously was also evaluated with a MWU test. In order to identify the type of consensus decision-making, we calculated Pearson's chi-squared tests with Yate's continuity correction (corrected for the different proportions of 1- and 5-bottle-platforms in designs 1 and 2) to compare the number of *non-fission* visits to platforms baited with 5 and 1 bottle(s), respectively. We used Fisher's exact tests to assess whether the study group preferred particular platforms, irrespective of baiting patterns in design 1 and 2. We also used Fisher's exact tests to determine whether overtaking increased with the number of bottles on the platform the movement was directed to (corrected for proportions of observed group movements towards platforms baited with 0, 1 and 5 bottles, respectively). To compare overtaking rates and initiatorship during movements to drinking platforms to observations of the same group, from the same time but from non-experimental contexts, we used chi-squared tests. To test whether the number of baited platforms (2 or 4) impacted fissioning, we used a chi-squared test. To analyze whether the group fissioned more often after visiting a 1- or a 5-bottle-platform (corrected for proportions of observed non-fission visits to platforms baited with 1 and 5 bottles in design 0/1 and 2, respectively), we used Fisher's exact tests. Tests on initiatorship and overtaking were run with PASW Statistics 18 (SPSS Inc., Chicago, IL, 2009), the other tests were run using R software (R Development Core Team, Vienna, Austria, R2.11.1).

In order to analyse determinants of resource intake and aggression, we ran three linear mixed models (LMM) and one generalized linear mixed model (GLMM; [54]). We chose these models because they allowed exploring influences of several explanatory variables simultaneously while controlling for repeated observations of the same individuals, as well as for artificial variance resulting from a variable number of bottles per platform. Individual identity and number of bottles per platform were fitted as crossed random factors. We chose number of bottles as random factor rather than total resource volume per platform because bottles represented the monopolizable units for the individuals. Furthermore, number of bottles and total resource volume per platform were highly correlated (Pearson correlation coefficient: n = 20; r = 0.62, p<0.01). Tables 2 and 3 provide an overview of data distribution, error structure and explanatory variables of the models. The first model in Table 2 explored whether departure order explained resource intake at a platform and was based on the observations of group movements towards the platforms. The second model was based on data from the video recordings and assessed the impact of arrival order, sex and age on resource intake, including visits where only one individual had been recorded at a platform. Additionally, we included visit number per day as fixed factor to account for repeated visits. In the first model in Table 3, we analysed which factors (sex, age, number of individuals, visit number, number of baited platforms) influenced aggression. We used count data of agonistic interactions (Poisson distribution with overdispersion, fitted with a quasi-Poisson GLMM) as response variable and controlled for the time spent on the platform as a fixed factor. The second model explored whether individual resource intake at the platforms was affected by the individual aggression rates, again taking individual age, sex and number of visits at the same platform per day into consideration. For both models on aggressiveness, we used only visits where ≥2 individuals were present at a platform.

**Table 2:** Parameter estimates for the most parsimonious linear mixed models (LMM) on determinants of resource intake.

Model	Response variable	Random factors	Fixed factors	Estimate	SE	P-value
**Predictors of resource intake**						
At departure, LMM (intakes of individuals observed during movements to platforms; n = 31)	Individual resource intake at platforms (sqrt %)	Animal ID, number of bottles per platform	Intercept	3.40	1.16	0.15
			Departure position (leader, follower)	−0.10	1.38	0.99
At arrival, LMM (intakes of all individuals arriving at platforms; n = 204)	Individual resource intake at platforms (sqrt %)	Animal ID, number of bottles per platform	Intercept	5.55	1.65	<0.01**
			Arrival order	−1.10	0.12	<0.001***
			Visit number per platform per day	−0.48	0.27	0.08
			Age (adult, subadult)	0.09	0.52	0.90
			Sex (female, male)	0.81	0.54	0.23

Significance level is at 0.05. sqrt = square-root-transformed data, ID = identity, SE = standard error.

**Table 3:** Parameter estimates for the most parsimonious (generalized) linear mixed model (GLMM and LMM) on determinants and benefits of aggressiveness.

Model	Response variable	Random factors	Fixed factors	Estimate	SE	P-value
**Aggressiveness on platforms**						
Determinants of aggressiveness, Quasi-Poisson GLMM (≥2 individuals per platform; n = 147)	Counts of aggressions emitted	Animal ID, number of bottles per platform	Intercept	-5.72	0.41	<0.001***
			Number of individuals on platform^1^	0.48	0.03	<0.001***
			Time on platform (log sec)	1.50	0.13	<0.001***
			Age (adult, subadult)	−1.36	0.25	<0.05*
			Visit number per platform per day	Not included in final model		
			Sex (female, male)	Not included in final model		
			Number of baited platforms	Not included in final model		
Benefits of aggressiveness, LMM (≥2 individuals per platform; n = 147)	Individual resource intake at platforms (sqrt %)	Animal ID, number of bottles per platform	Intercept	2.49	1.20	0.25
			Individual aggression rates at platforms (events/hour)	−0.00	0.01	0.66
			Visit number per platform per day	0.10	0.32	0.74
			Sex (female, male)	0.50	1.15	0.58
			Age (adult, subadult)	0.49	1.11	0.65

Significance level is at 0.05. log = log-transformed data, sqrt = square-root-transformed data, ID = identity, SE = standard error.

1 Number of group members that were present on the platform ≥50% of the time with the individual. Times that an individual spent on the platform alone were excluded from the analysis.

For models with normal error structure, we used Akaike's Information Criterion [55] to remove parameters in a step-wise fashion in order to select the most parsimonious model with the best fit. Factors were excluded only if this improved the model fit by >2 AIC units [56], [57]. We used maximum likelihood ratio tests to test whether a fixed factor explained a significant amount of the variance in the presence of the other factors and to test the final model with fixed factors against the null model including only the random factors [58]. In case of overdispersion, we corrected standard errors using a quasi-GLM-model [54]. Because AIC calculations were not available for this model, we used chi-squared tests with the deviances of two models differing in only one fixed factor to select the most parsimonious model [58]. We used square-root and log-transformation on some variables to improve the model fit [54]. The significance level was set at 0.05.

## Results

### General responses to the experimental setup

**Figure 2:**
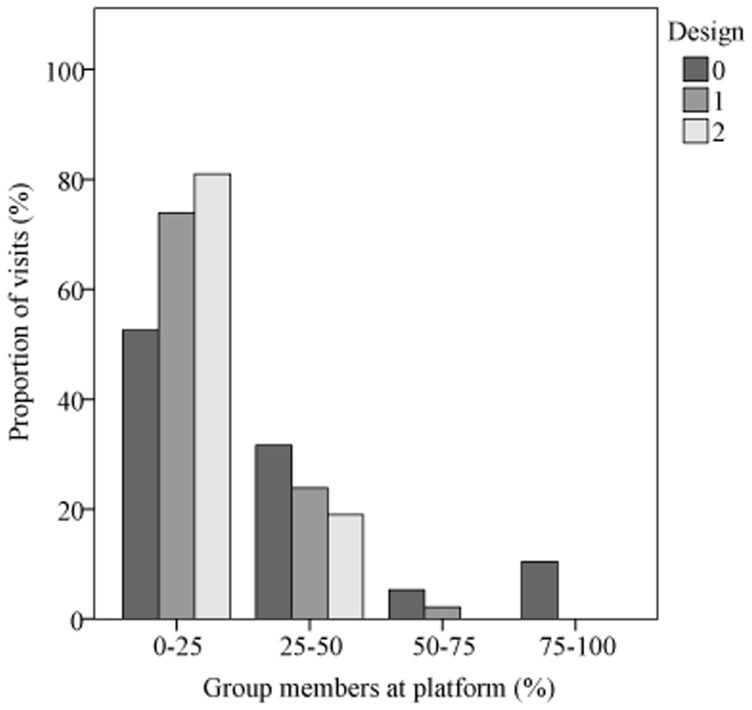
Proportion of group members (in percent) that was visible on the video recordings during platform visits throughout different experimental designs (*N* = 110).

All baited platforms were visited frequently throughout the experiment with individual platforms being visited 0–4 times per day (Table S1). Each group member visited the 4 platforms and drank more than 10 times in total, except for 2 adult males (BMGig: 3 visits; BMNeg: 8 visits) and 1 subadult male (BMRut: 9 visits). Individuals arriving at a platform emptied at once the 75 ml-bottles in 44%, the 30 ml-bottles in 84% and the 10 ml-bottles in 100% of visits. Only rarely were more than 25–50% of group members visible at the platforms on the video recordings (Figure 2). However, the number of individuals recorded at a platform increased significantly with the number of bottles per platform (MWU test: 1 bottle: n = 61, 2.4±1.8; 5 bottles: n = 39, 3.5±2.2; U = 798.0, z = −2.76, p<0.01).

### Consensus decision-making

The number of *non-fission* visits to platforms baited with 5 bottles compared to platforms baited with only 1 bottle did not differ from random expectations in either design (chi-squared tests: design 1: n = 21; χ^2^ = 0.01, df = 1, p = 0.93; design 2: n = 27; χ^2^ = 0.02, df = 1, p = 0.89). Therefore, we were not able to assess whether consensus decision-making was shared or unshared based on the decision outcomes observed during the experiment. In fact, the group visited platform 3 more often than the other platforms in each condition, i.e. independent of baiting patterns (non-fission visits to baited platforms). However, differences between the visit numbers at platforms were not significant compared to random expectations (condition 1–3: 25% visits per platform expected; conditions 4–5: 50% visits for platforms 2 and 3 expected; Fisher's exact tests: condition 1: n = 13; p = 0.11; condition 2: n = 9; p = 0.62; condition 3: n = 13; p = 0.41; condition 4: n = 11; p = 0.68; condition 5: n = 16; p = 0.72).

### Group fissions

The group split into subgroups 21 times comprising a total of 41 visits at platforms (not 42 because sometimes more than 2 subgroups formed and some visits were part of two different fission events). There were 69 non-fission visits at platforms. If a fission event (n = 21) - when individuals visit different platforms at the same time - is counted as a single visit, fission events make up 23.3% of all visits. We witnessed 6 fission events directly during the 25 movements to platforms that we observed (24%), i.e. we observed movements that resulted in group members being recorded at two different platforms at the same time or <1 min after one another. Two subgroups were recorded at different platforms on 18 occurrences,3 subgroups on two occurrences. Subgroup size ranged from 1 to 5, with a mean of 2.0±1.1 individuals. The proportion of group fissions compared to non-fission visits was larger when 4 platforms were baited (31.4%) instead of only 2 platforms (12.8%) but the difference was not statistically significant (chi-squared test: n = 90, fissions counted as one event; χ^2^ = 3.28, df = 1, p = 0.07). The group did not split into subgroups more often after visiting a 1- compared to a 5-bottle-platform in the experimental designs 0/1 (Fisher's exact tests,: n_non-fission_ = 34; n_fission_ = 5, p = 1.00).The sample size dropped for this test because we had to exclude fission visits and visits to non-baited platforms, as well as fission events occurring during the first visits recorded on a day or following immediately after another fission event. Therefore the number of suitable fissions observed during the experimental design 2 precluded any statistical analysis (n_non-fission_ = 27; n_fission_ = 1). However, during non-fission visits (n_non-fission_ = 69) on average less individuals (1.1±1.2) drank simultaneously than during fission visits (n_fission_ = 17, 1.8±1.2 individuals; MWU: U = 387.5, z = -2.15, p<0.05).We excluded the 4 fission events during which individuals did not *stay* on different platforms at the same time but arrived at different platforms less than 1 min after one another because they were not able to drink simultaneously. It is not feasible to extend the calculation to individuals *drinking* <1 min time delayed, either, because individuals often stayed on the platforms for some time after depleting the bottles. Thus, usually no animal drank during the last minutes on a platform.

### Group coordination, overtaking and resource intake

**Figure 3:**
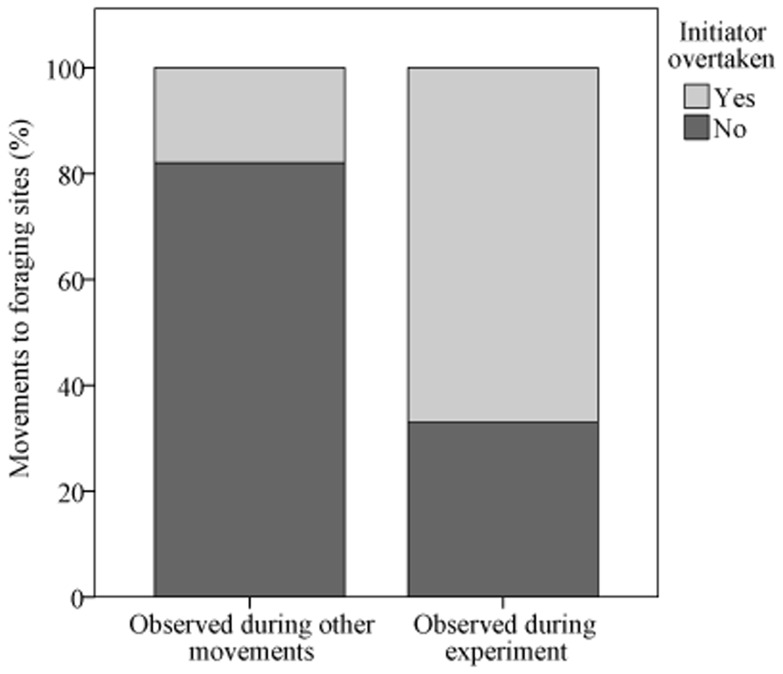
Overtaking events (in percent) during movements towards drinking platforms compared to other foraging movements of the group observed during the same time period. *N* = 15 observations of group movements for the experiment, *N* = 57 observations of group movements for other observations.

**Figure 4:**
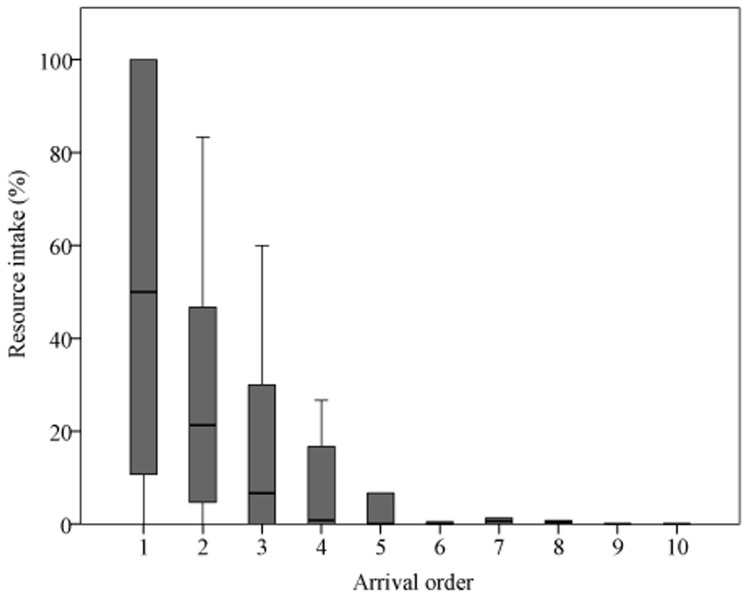
Individual resource intake (in percent of total volume available at the platform) in relation to arrival order. Shown are medians, 25–75% quartiles (box) and ranges (whiskers). *N* = 204.

Observed movements towards drinking stations (n_total_ = 25; n_with followers_ = 15) were initiated by adults of both sexes and by one subadult male, with the oldest female of the group being responsible for 72% of all initiations. This high proportion of female initiatorship did not differ from observations of natural group movements of the same group ([45]; chi-squared test: n_exp_ = 25, n_other_ = 251; χ^2^ = 0.11, df = 1, p = 0.74). However, overtaking rates during the experiment (67%) were significantly higher than expected from movements not directed at foraging platforms of the same group during the same time period (18%; chi-squared test: n_exp_ = 15, n_other_ = 57; χ^2^ = 47.1, df = 1, p<0.001; Figure 3). We observed individuals of all sex and age classes overtaking throughout all conditions. However, overtaking rate did not decrease with the number of bottles on the platform visited (Fisher's exact test: n = 10; p = 0.37). Departure position, i.e. whether an individual initiated or followed a movement, did not affect individual resource intake (Table 2) since the full model did not explain significantly more variance than the null model (LMM: n = 31; χ^2^ = 0.00, df = 1, p = 0.95). In contrast, arriving first was advantageous in terms of foraging benefits (Table 2; Figure 4), and the model differed significantly from the null model (LMM: n = 204; χ^2^ = 61.30, df = 4, p<0.001). Visit number per platform per day, sex and age were part of the final model but did not contribute significantly (Table 2).

### Social interactions at the platforms

Agonistic interactions (N = 111) occurred during 30 out of 54 visits during which ≥2 individuals were present at a baited platform (55.6%). In 8 cases (7.2%), a single agonistic action (bite, cuff, grab) resulted in the direct displacement of a conspecific from a bottle and offered a drinking opportunity for the aggressor. Individuals that had previously overtaken the leader were never displaced from a bottle. Aggressive interactions were more likely to happen when more individuals were present at a platform and when an individual spent more time at the platform (Table 3). Furthermore, adults showed more aggressive behavior than subadults. In contrast, the sex of the individuals, the number of visits per day and the number of baited platforms did not contribute to the most parsimonious model (Table 3). The final model performed significantly better than the null model (quasi-Poisson GLMM: n = 147 individual visits with ≥2 individuals; χ^2^ = 53.33, df = 3, p<0.001). However, a higher aggression rate did not result in increased resource intake. The other factors of the most parsimonious model, visit number, age and sex, did not contribute significantly, either. The final model did not have a better fit than the null model (LMM: n = 147 individual visits with ≥2 individuals; χ^2^ = 0.75, df = 4, p = 0.94).

## Discussion

As previous field experiments with single primate groups [31], [33], [34] our study provides important insights into the diversity of coordination processes and related consequences for individual foraging benefits. Redfronted lemurs reacted to changes in experimental design in terms of more individuals visiting the platforms when more bottles were provided. However, the number of visits per platform did not depend on baiting patterns. Therefore, we were not able to identify the decision type underlying the observed outcomes. Adult females initiated most movements towards the platforms. However, in contrast to observations in natural contexts, change of leadership was more frequent so that initiators did not enjoy higher resource intake than followers. Group fissions resulted in more individuals drinking simultaneously. Fissioning was not affected by changes in baiting patterns, however. Individual aggression rates did not affect foraging benefits in terms of higher resource intake. We discuss each of these findings in more detail below.

### General responses to the experimental setup

Most individuals of our study group visited the platforms and depleted the experimental resource regularly, which was a prerequisite for the foraging experiment and reflects the resource value. The proportions of different resource volumes depleted by single individuals during the experiment were similar to those assessed during the pilot study. However, usually only a few group members were visible at the platforms on the video recordings. This could be a result of group fissions during which only one subgroup visited a platform. Unfortunately, we did not capture these fission events with our definition that relied on two subgroups being video-recorded at different platforms at the same time or <1 min after one another. However, the number of individuals recorded increased significantly with the number of bottles at the platforms, which might indicate that the group was usually present around the platform and a larger number of group members only jumped onto the platform, i.e. inside the camera frame, when more than one bottle was present. It also shows that more bottles provided indeed more individuals with a drinking opportunity, i.e. single individuals did not monopolize entire platforms.

### Consensus decision-making

We tried to identify whether decision-making in groups of redfronted lemurs was shared or unshared using platforms that provided either one or several group members with a foraging opportunity. However, the number of group visits to a platform was not obviously affected by baiting patterns, and we were not able to identify the decision type underlying the observed decision outcomes. There are several reasons that may explain this result.

1. It was difficult to determine the resource volume that offered a sufficient foraging benefit for exactly one individual. For instance, the random visiting patterns observed in design 2 could be due to unshared decision-making if the decision-maker always drank more than 30 ml, i.e. led the group also to the 5-bottle-platform after depleting the 1-bottle-platform. Furthermore, we offered a maximum of 5 foraging opportunities. However, decision-making might be *partially* shared with only 2–3 individuals contributing to the outcome (e.g., [15], [16]). Under these circumstances, a 1-bottle-platform offering 75 ml could have provided foraging benefits for all individuals involved in the decision-making.
2. The concept of discrete, monopolizable resource units (bottles) that have the same size but offer different foraging benefits could lack ecological relevance because redfronted lemurs usually feed on dispersed fruit and leaves [59], [60].
3. Our experimental setup comprising four platforms was quite complex. Therefore, the lemurs may have failed to react towards variation in baiting patterns because we changed between different conditions too fast.
4. We routinely refilled bottles in the afternoon and often the study group visited platforms even more than 2 times per day due to refilling after depletion by a group that had not been observed. Maybe the total amount of flavored water available was, therefore, more than enough for the whole group.
5. Different platforms could be visited by the same individual subsequently in short time intervals (as little as 64 sec). This might have reduced the incentive for consensus decision-making.

Alternatively, it is also possible that consensus decision-making does not present the only means to optimize group foraging benefits. For instance, group members can avoid consensus costs through different strategies such as (1) fissioning, which can presumably maximize the benefits of a majority of individuals (see e.g., [10]), (2) shifting the benefits of consensus decision-making by overtaking initiators or aggressively displacing group members that arrived earlier at the resource. We tried to identify the determinants and outcomes of each of these possibilities and subsequently discuss the respective results.

### Group fissions

Regarding determinants of group fissions, we predicted that fission probability would increase with the number of baited platforms and after the group visited a poorly baited platform. Neither of these hypotheses was supported, suggesting that baiting patterns did not affect group fissions. However, we cannot exclude the possibility that our operational definition of fission, which was constrained by the use of automatic cameras, might have captured only a subset of the real fission events. We could not compare fission rates during the experiment ( = inter-individual distances of 75 m) to natural contexts because respective data on inter-individual distances of >20 m under natural conditions are lacking. However, even given the possible shortcomings of our definition of a fission event, it is remarkable that individuals relaxed group cohesion and different subgroups exploited different platforms at the same time in 23.3% of the visits although platforms could be exploited subsequently in short time intervals (64 sec).

In accordance with our third prediction, fissioning resulted in a significantly higher number of individuals drinking from bottles at the same time. Hence, fissioning apparently allowed group members to avoid costly consensus decisions and provided instant foraging benefits for a higher number of individuals [10]. Such temporary splitting into subgroups as a consequence of high consensus costs has been observed in a number of species, including pigeons [11], bats [12] and baboons [13]. However, there are also potential costs of group fissions. Decreased cohesion should negatively affect shared vigilance and predator confusion and, thus, increase predation risk [1], [3]. This may be particularly salient during the dry season when we conducted the experiment because during this time the fossa mainly feeds on lemurs [61], and predation risk is supposed to be high due to defoliated trees [62]. Thus, benefits of group fissions in terms of enhanced resource access supposedly outweighed the costs of increased predation risk during the experiment (see also [63], [64]).

### Group coordination, overtaking and resource intake

We predicted predominantly stable leadership by adult females towards the platforms and, accordingly, highest foraging benefits for these individuals. Consistent with our predictions, adult females initiated most movements towards the platforms. Female initiatorship and leadership have also been described in other species (dwarf mongooses: [37]; Grevy's zebras, *Equus grevyi*, and onagers, *Equus hemionus*: [65]; plains zebras: [38]; primates: summarized in [43]) and is usually attributed to the increased energetic needs of females during gestation and lactation. Since we conducted the experiment during the gestation period of redfronted lemurs, females might have initiated group movements in order to compensate increased physiological requirements as well.

However, female leadership was not stable. In fact, overtaking by individuals of all age and sex classes occurred during two-thirds of movements towards experimental platforms. During natural movements of the same study population, change of leadership occurred in only 18% of cases (this study) and 9.4% in another study [45]. No overtaking at all was reported in another study on group movements in redfronted lemurs [44]. As a consequence of overtaking, initiators did not arrive first at the drinking platforms, and arrival instead of departure order predicted individual foraging benefits. Neither sex nor age affected resource intake, and it was not always the same individuals who arrived at a platform first. We expected higher overtaking rates during movements towards 1-bottle-platforms. However, the number of bottles did not influence change of leadership, which is in line with the observation that baiting patterns did not affect visit numbers at platforms, either.

Overtaking rates in the present study were apparently affected by the high value and predictability of the experimental resource compared to natural foraging patches. However, such changes in coordination processes have not been described in comparable studies involving movements to experimental foraging patches by groups of white-faced capuchin monkeys [29] and chacma baboons [13]. These species are characterized by clear dominance hierarchies that have been shown to result in asymmetric resource-holding potential [47], [66]. Therefore, the high frequency of overtaking by individuals from all age and sex classes observed here could be facilitated by the egalitarian social relationships among redfronted lemurs [35], [36]. In fact, we never observed an individual that had previously overtaken being threatened by other group members after arrival on the platforms.

### Social interactions at the platforms

A third tactic to optimize individual foraging benefit could be supplanting of group members from bottles through aggressive behavior at the platforms. As in other studies on redfronted lemurs [44], [67], we observed aggressive behavior during feeding throughout the experiment. Aggression increased with the number of individuals and the time spent on the platform. Furthermore, adults emitted aggression more often than subadults. However, contrary to our hypothesis, the frequency of agonistic interactions at the platforms was not higher on platforms with fewer bottles.

At first sight, higher aggression levels of adults compared to subadults seem to reflect the proximate importance of physical asymmetry in accessing a food resource [68], [69]. However, aggression resulted in the displacement of a conspecific from a bottle in only 7.2%. Therefore, aggression was not a powerful mechanism to obtain a foraging opportunity but rather a consequence of increased agitation or opportunities for cuffs and grabs due to spatial crowding [70]. The assumption of high social tolerance exhibited by adults towards subadults at the platforms is also in line with another observation: mothers and their offspring were observed to drink simultaneously from the same bottle for prolonged time spans (>5 sec) in 22 cases.

### Conclusions

Our study permits two more general conclusions regarding the relationships among coordination, foraging behavior and social structure in socially structured groups. First, costs of a temporary lack of coordinated cohesion can be outweighed by high resource quality and predictability, even under supposedly high predation risk. Second, coordination in animal groups characterized by an egalitarian social structure can be highly flexible, allowing the use of alternative behavioral tactics that translate into individual foraging benefits irrespective of sex, age or social status.

## Supporting Information

Table S1 **Number of visits of the study group on single platforms per study day throughout different conditions/designs.**
(DOCX)
