# Supplementary material for: Determinants and Outcomes of Decision-Making, Group Coordination and Social Interactions during a Foraging Experiment in a Wild Primate
Source: PLoS One. 2013 Jan 10;8(1):e53144. doi: 10.1371/journal.pone.0053144 (PMC3542333; doi:10.1371/journal.pone.0053144)
Supplement: Table S1 — Number of visits of the study group on single platforms per study day throughout different conditions/designs. (DOCX) [file pone.0053144.s001.docx]

| **Design** | **Condition** | **Day of experiment** | **Platform number** | | | |  |
| --- | --- | --- | --- | --- | --- | --- | --- |
|  |  |  | **1** | **2** | **3** | **4** |  |
| **0** | **1** | **1** | 4 | 3 | 4 | 0 | |
|  |  | **2** | 0 | 1 | 0 | 0 | |
|  |  | **3** | 1 | 1 | 3 | 0 | |
|  |  | **4** | 0 | 0 | 2 | 0 | |
| **1** | **2** | **5** | 1 | 1 | 2 | 1 | |
|  |  | **6** | 2 | 0 | 1 | 0 | |
|  |  | **7** | 2 | 1 | 3 | 0 | |
|  |  | **8** | 2 | 1 | 2 | 1 | |
|  | **3** | **9** | 0 | 1 | 3 | 0 | |
|  |  | **10** | 2 | 1 | 4 | 3 | |
|  |  | **11** | 2 | 1 | 3 | 4 | |
|  |  | **12** | 1 | 1 | 1 | 1 | |
| **2** | **4** | **13** | 0 | 1 | 2 | 2 | |
|  |  | **14** | 2 | 3 | 2 | 0 | |
|  |  | **15** | 0 | 1 | 1 | 1 | |
|  |  | **16** | 0 | 1 | 3 | 0 | |
|  | **5** | **17** | 1 | 3 | 2 | 0 | |
|  |  | **18** | 1 | 0 | 4 | 1 | |
|  |  | **19** | 0 | 3 | 4 | 2 | |
|  |  | **20** | 0 | 1 | 2 | 0 | |
